# Hepatic Sclerosed Hemangioma: a case report and review of the literature

**DOI:** 10.1186/s12893-015-0029-x

**Published:** 2015-04-17

**Authors:** Shunsuke Miyamoto, Akihiko Oshita, Yutaka Daimaru, Masaru Sasaki, Hideki Ohdan, Atsushi Nakamitsu

**Affiliations:** Department of Surgery, JA Hiroshima General Hospital, 1-3-3, Jigozen, 738-8503, Hatsukaichi, Japan; Department of Gastroenterological and Transplant Surgery, Applied Life Sciences, Institute of Biomedical and Health Sciences, Hiroshima University, Hiroshima, Japan; Department of Pathology, JA Hiroshima General Hospital, Hatsukaichi, Japan

**Keywords:** Hepatic, Sclerosed, Hemangioma, US, CT, MRI, FDG-PET

## Abstract

**Background:**

Although cavernous hemangioma is one of the most frequently encountered benign hepatic neoplasms, hepatic sclerosed hemangioma is very rare. We report a case of hepatic sclerosed hemangioma that was difficult to distinguish from an intrahepatic cholangiocarcinoma by imaging studies.

**Case presentation:**

A 76-year-old male patient with right hypochondralgia was referred to our hospital. Abdominal ultrasonography revealed a heterogeneously hyperechoic tumor that was 59 mm in diameter in segment 7 of the liver. Dynamic computed tomography showed a low-density tumor with delayed ring enhancement. Gadolinium-ethoxybenzyl-diethylenetriamine pentaacetic acid-enhanced magnetic resonance imaging (EOB-MRI) demonstrated a low-signal intensity mass with ring enhancement on T1-weighted images. The mass had several high-signal intensity lesions on T2-weighted images. EOB-MRI revealed a hypointense nodule on the hepatobiliary phase. From these imaging studies, the tumor was diagnosed as intrahepatic cholangiocarcinoma, and we performed laparoscopy-assisted posterior sectionectomy of the liver with lymph node dissection in the hepatoduodenal ligament. Histopathological examination revealed a hepatic sclerosed hemangioma with hyalinized tissue and collagen fibers.

**Conclusion:**

Hepatic sclerosed hemangioma is difficult to diagnose preoperatively because of its various imaging findings. We report a case of hepatic sclerosed hemangioma and review the literatures, especially those concerning imaging findings.

## Background

The preoperative diagnosis of hepatic sclerosed hemangioma is very difficult, even with recent developments in radiological modalities, because it is an extremely rare benign disorder and its radiological features resemble those of hepatic malignancies such as cholangiocarcinoma and metastatic liver cancer [1,2]. We report a case of a hepatic sclerosed hemangioma, that had been preoperatively misdiagnosed as an intrahepatic cholangiocarcinoma and been resected, and review the relevant literature, especially summarizing the imaging findings of hepatic sclerosed hemaigioma.

## Case presentation

A 76-year-old male patient had consulted a doctor for upper abdominal pain 16 months before being referred to us and had been followed up. Because plain computed tomography (CT) revealed a space-occupying lesion in the liver, he was referred to our hospital. A laboratory workup on admission showed that total bilirubin, aspartate aminotransferase, alanine aminotransferase, alkaline phosphatase, gamma-glutamyl transpeptidase, albumin, and creatinine were all within normal ranges. Tumor markers including alpha-fetoprotein, protein induced by vitamin K absence or antagonist-II, carcinoembryonic antigen, and carbohydrate antigens 19–9 were also within the normal limits (Table 1).

**Table 1:** **Review of imaging features for Hepatic Sclerosed Hamangioma**

**Case**	**Year**	**Author**	**Age**	**Gender**	**Location**	**Size (mm)**	**US**	**Plain CT**	**Dynamic CT**	**MRI (T1/T2)**	**PET-CT**	**Preoperative diagnosis**	**Final diagnosis**
1	1995	Ishii	81	F	S3	25	Low	Low	Ring enhanced	Low/Low	―	Not determined	sclerosed hemangioma
2	1995	Haratake	64	F	S8	26	―	Low	Ring enhanced	―	―	Metastatic liver cancer	sclerosed hemangioma
3	1996	Kobayashi	49	F	S7	22	High	Low	Ring enhanced	Low/High	―	Not determined	sclerosed hemangioma
4	1998	Ukai	66	F	S6	10	Low	Low	Ring enhanced	Low/High	―	Hepatocellular carcinoma	sclerosed hemangioma
5	2000	Yamashita	67	F	S4	50	Low	Low	Ring enhanced	High/High	―	Metastatic liver cancer	sclerosing hemangioma
6	2001	Okada	77	M	S8	23	High	Low	Not enhanced	―	―	Metastatic liver cancer	sclerosed hemangioma
7	2001	Aibe	67	F	S4	40	―	Low	Not enhanced	High/High	―	Metastatic liver cancer	sclerosed hemangioma
8	2003	Hayakawa	70	F	S2	30	Low	Low	Ring enhanced	Low/Iso-High	―	Not determined	sclerosed hemangioma
9	2005	Morikawa	66	M	S8	50	Iso	Low	Ring enhanced	Low/High	Not accumulated	Not determined	sclerosed hemangioma
10	2005	Lee	65	F	S6	53	―	―	Ring enhanced	Low/High	―	Hepatocellular carcinoma	sclerosing hemangioma
11	2005	Okamoto	50	F	S3	30	Low	Low	Ring enhanced	―	―	sclerosed hemangioma	sclerosed hemangioma
12	2006	Hamatsu	59	M	S8	25	High	Low	Ring enhanced	―	―	Metastatic liver cancer	sclerosed hemangioma
13	2006	Hayashi	82	F	S2/3	55	High	Low	Not enhanced	Low/High	―	Gastric submucosal tumor	sclerosed hemangioma
14	2006	Iida	77	F	S2	39	High	Low	Ring enhanced	Low/Low-High	Not accumulated	Not determined	sclerosing hemaigioma
15	2007	Sawai	67	F	Right robe	145	Low	Low	Ring enhanced	Low/High	―	Not determined	sclerosed hemangioma
16	2008	Kaji	65	F	S5	25	Low	Low	Ring enhanced	Low/Iso-High	―	Cholangiocarcinoma	sclerosed hemangioma
17	2008	Tsumaki	70	F	S8	47	Low	Low	Ring enhanced	Low/High	―	Liver sclerosed hemangioma	sclerosed hemangioma
18	2008	Mori	77	F	S6	100	High	Low	Not enhanced	Low/High	―	Cholangiocarcinoma	sclerosed hemangioma
19	2010	Yoshida	75	F	S5/6	37	High	Low	Ring enhanced	Low/High	Not accumulated	Cholangiocarcinoma	sclerosing hemaigioma
20	2010	Usui	57	F	S2	17	Low	Low	Ring enhanced	Low/High	―	Metastatic liver cancer	sclerosed hemangioma
21	2010	Jin	52	M	S6/7	38	―	―	Ring enhanced	Low/High	―	Hepatocellular carcinoma	sclerosed hemangioma
22	2010	Hida	75	F	S5/6	30	High	―	Ring enhanced	Low/High	―	Metastatic liver cancer	sclerosed hemangioma
23	2011	Miyaki	60’s	F	S3	30	Low	Low	―	Low/High	―	Liver sclerosed hemangioma	sclerosed hemangioma
24	2011	Kitami	72	F	S3	55	Low	Low	Ring enhanced	Low/High	―	Cholangiocarcinoma	sclerosed hemangioma
25	2011	Tanaka	71	M	S6	15	High	Low	Ring enhanced	―	―	Hepatocellular carcinoma	sclerosed hemangioma
26	2011	Mikami	74	F	S2	22	Low	Low	Ring enhanced	Low/High	Not accumulated	Not determined	sclerosed hemangioma
27	2011	Shin	50	M	Right robe	100	Iso-Low	Low	Ring enhanced	Low/High	Not accumulated	Liver sclerosing hemangioma	sclerosing hemangioma
28	2012	Wakasugi	61	F	S2, S5	25,5	Low	―	Ring enhanced	Low/High	―	Metastatic liver cancer	sclerosed hemangioma
29	2012	Yamada	75	M	S8	11	―	Low	Ring enhanced	Low/High	Not accumulated	Metastatic liver cancer	sclerosed hemangioma
30	2013	Song	63	F	S2/3	91	―	Low	Ring enhanced	―	―	Not determined	sclerosing hemangioma
31	2013	Shimada	63	M	S8	10	―	Low	Ring enhanced	Low/High	―	Atypical hemangioma	sclerosed hemangioma
32	2015	OUR CASE	76	M	S6/7	59	High	Low	Ring enhanced	Low/High	―	Cholangiocarcinoma	sclerosed hemangioma

**Figure 1:**
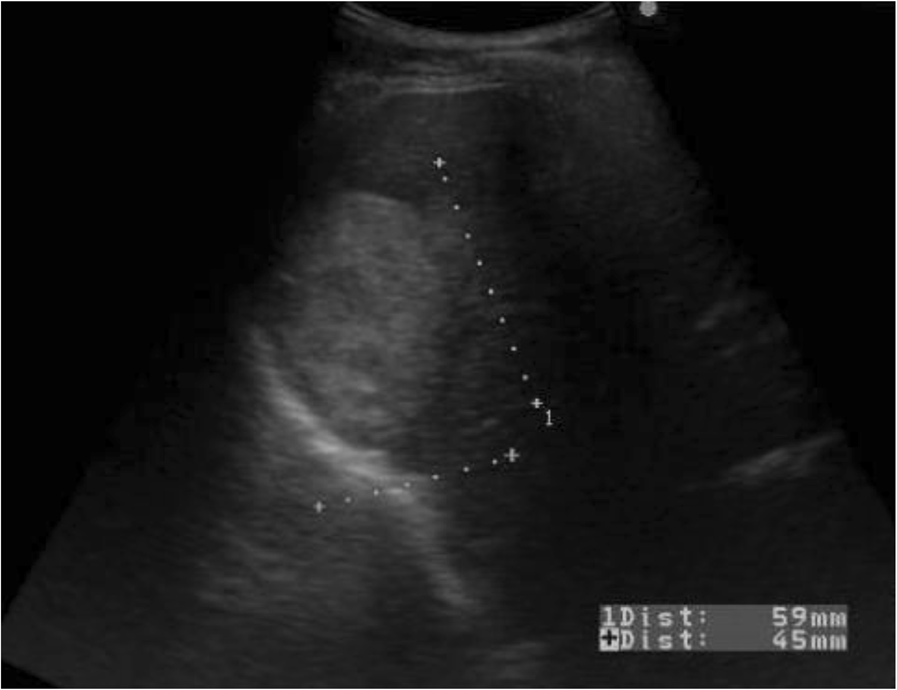
Abdominal ultrasonography (US). US showed a heterogeneously hyperechoic mass in segment 7 of the liver.

**Figure 2:**
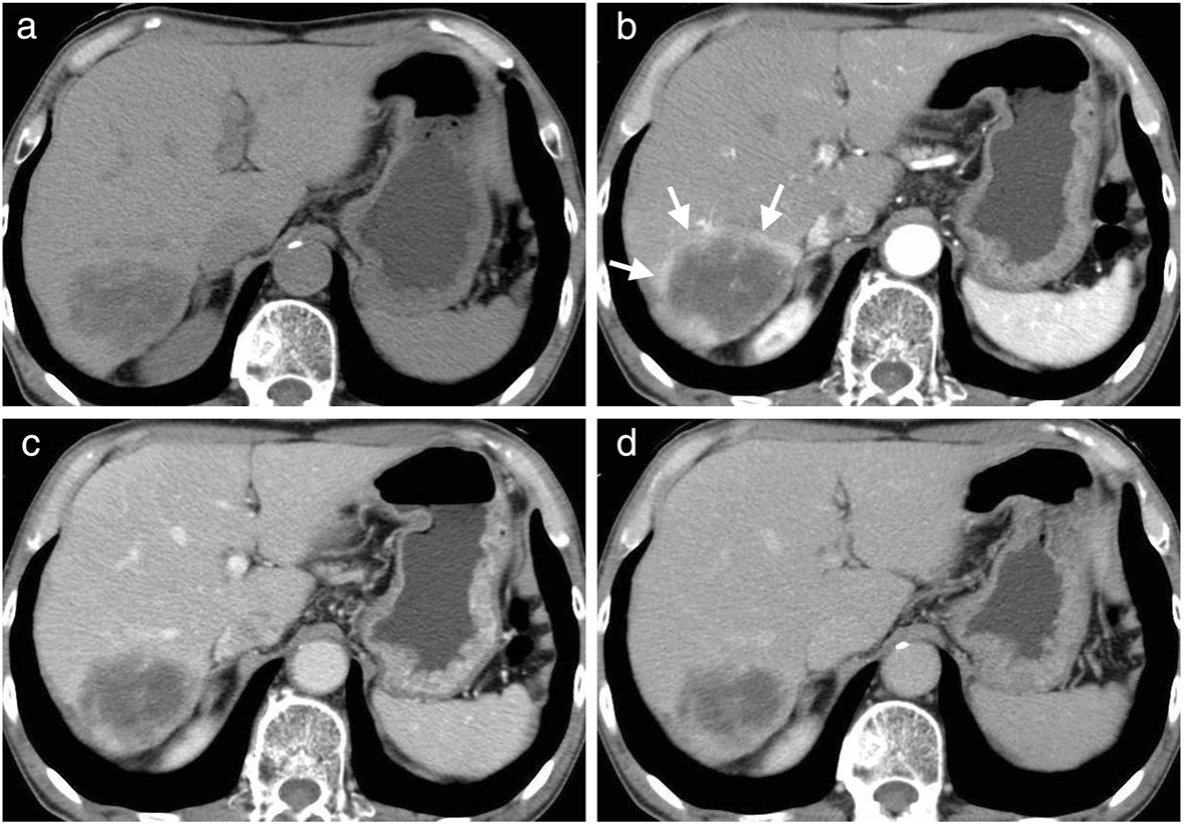
Abdominal computed tomography (CT). **(a)** plain, **(b)** arterial phase, **(c)** portal venous phase, and **(d)** delayed phase. Plain CT showed a low-density mass. Dynamic CT showed the ring enhancement in the peripheral part on the arterial phase (arrow).

**Figure 3:**
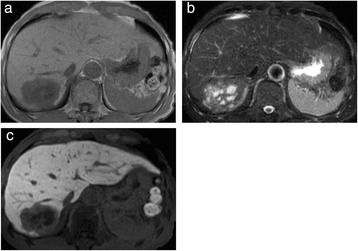
Magnetic resonance imaging (MRI). **(a)** T1-weighted image, **(b)** T2-weighted image, and **(c)** ethoxybenzyl (EOB)-MRI on the hepatobiliary phase. The tumor had low-signal intensity on T1-weighted and some high-signal intensity nodules in the tumor on T2-weighted images. EOB-MRI showed no uptake in the corresponding area on the hepatobiliary phase.

Abdominal ultrasonography (US) revealed a well-defined, heterogeneously hyperechoic mass that was 59 mm in diameter in segment 7 of the liver (Figure 1). Plain CT revealed a low-density 60-mm sized mass with an irregular margin. Dynamic CT revealed early ring enhancement in the peripheral part on the arterial phase and internal heterogeneous enhancement on the delayed phase (Figure 2). Gadolinium-ethoxybenzyl-diethylenetriamine pentaacetic acid-enhanced magnetic resonance imaging (EOB-MRI) showed that the tumor had low-signal intensity on T1-weighted images and that the mass had some high-signal intensity foci in the tumor on T2-weighted images. EOB-MRI showed no uptake in the corresponding area on the hepatobiliary phase and ring enhancement in the peripheral part on the arterial phase and the portal phase (Figure 3).

**Figure 4:**
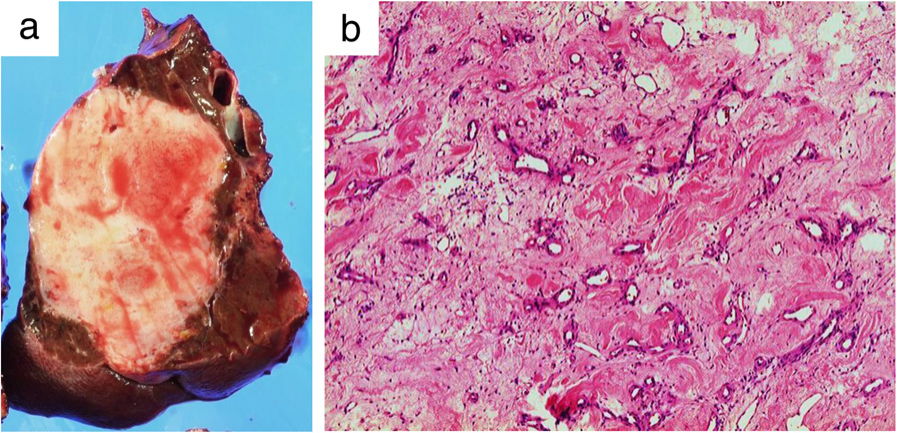
Resected specimen. **(a)** Surface of slice. The cut surface of the tumor reveals a white solid mass (61 × 46 mm in size) that was elastic, soft, and homogeneous with a smooth margin including some faint red spots, sized 1 cm. **(b)** Hematoxylin and eosin staining (magnification, ×100). The tumor was composed of fibrous connective tissue highlighted with collagen fiber and various sizes of cavernous hemangioma tissue with some hyaline degeneration secondary to thrombus, necrosis, or cicatrization.

Laparoscopy-assisted posterior sectionectomy and cholecystectomy including lymph node dissection in the hepatoduodenal ligament were performed for a preoperative diagnosis of intrahepatic cholangiocarcinoma. The resected specimen revealed a white solid mass, sized 61 × 46 mm. The cut surface of the tumor was elastic, soft, and homogeneous with the smooth margin including some faint red spots up to 10 mm in size (Figure 4a).

Histopathological examination showed that the tumor was composed of fibrous connective tissue highlighted with collagen fibers and various sizes of cavernous hemangioma tissue with some hyaline degeneration secondary to thrombus, necrosis, or cicatrization, resulting in a hepatic sclerosed hemangioma (Figure 4b).

The postoperative course was uneventful. The patient was discharged on postoperative day 6.

## Discussion

Hepatic sclerosed hemangioma, first reported by Ishii in 1995 [1], is a rare disease, detected and reported in only 2 out of 1000 cases on autopsy [3]. We found only 9 cases in PubMed by manual searching for the terms “hepatic, sclerosed, hemangioma” and “hepatic, sclerosing, hemangioma” from January 1983 to January 2015. Additionally, we found 22 cases in ICHUSHI, a bibliographic database established in 1903 and being updated by the Japan Medical Abstracts Society, contains bibliographic citations and abstracts from more than 2500 biomedical journals and other serial publications published in Japanese. The 32 cases, including our case, are summarized in Table 1 [1,4-33].

Hepatic sclerosed hemangioma is caused by degenerative changes such as thrombus formation, necrosis, and scar formation of liver cavernous hemangioma, but the mechanism for degenerative changes in the hepatic cavernous hemangioma has not been well clarified at present [34].

Concerning the imaging studies, Doyle et al. summarized the imaging findings of 10 hepatic sclerosed hemangioma lesions and found the characteristic features to include a geographic pattern, capsular retraction, decrease in size over time, loss of previously seen regions of enhancement [2]. And additional characteristic, features included the presence of transient hepatic attenuation difference, ring enhancement, and nodular regions of intense enhancement as seen in typical hemangioma. In our series reviewed the average size of the hepatic sclerosed hemangiomas was 42.3 mm, ranging from 10 to 145 mm. Abdominal US showed a hyperechoic mass in 11 cases and a hypoechoic tumor in 13 cases. Plain CT was likely to show a low-density mass, and dynamic CT showed ring enhancement, resembling metastatic liver cancer or intrahepatic cholangiocarcinoma, in 27 of 31 reported cases. MRI showed a low-intensity signal in 24 of 26 reported cases on T1-weighted images and a high-intensity signal in 22 of 26 reported cases on T2-weighted images. The radiological features revealed by dynamic CT and MRI resembled those of hepatic malignancies, leading to preoperative misdiagnosis. Whereas, [^18^F]-fluorodeoxyglucose positron emission tomography (FDG-PET), performed in just 6 cases, showed no accumulation of [^18^F]-FDG (Table 1). FDG-PET could be helpful in preoperative diagnosis to distinguish benign sclerosed hemangioma from malignant tumors such as intrahepatic cholangiocarcinomas or metastatic liver cancers. We may have had to perform FDG-PET preoperatively.

Surgical resection for hepatic sclerosed hemangioma is controversial. Most of the tumors reported were resected due to a preoperative misdiagnosis of malignancy (Table 1). To make a definite diagnosis of such hepatic tumors, percutaneous needle biopsy is not acceptable because of the possibility of dissemination of the cancer cells if the tumor is malignant. Therefore we would suggest that hepatic resections are chosen for the management of hepatic sclerosed hemangioma at present.

Makhlouf and Ishak compared the findings of sclerosed hemangioma and sclerosing cavernous hemangioma. According to their theory, recent hemorrhages and hemosiderin deposits rich in mast cells are present in the sclerosing hemangioma. While, fibrosis, increased elastic fibers, and dystrophic or psammomatous calcifications with a decreased number of mast cells can be observed in the sclerosed hemangioma [35]. Our case showed a fibrous connective tissue highlighted with collagen fibers and various sizes of cavernous hemangioma tissue with some hyaline degeneration. These findings are consistent with features of hepatic sclerosed hemangioma, resulting in the final diagnosis.

## Conclusion

We report a case with a hepatic sclerosed hemangioma. Although it is a rare disease, it is important to distinguish hepatic sclerosed hemangioma from hepatic malignancies. However, it is extremely difficult to diagnose precisely from imaging studies. If the possibility of a malignant tumor cannot be ruled out, hepatic resection might be selected for diagnostic therapy.

## Consent

Written informed consent was obtained from the patient for publication of this case report and any accompanying images. A copy of the written consent is available for review by the Editor of this journal.

## Abbreviations

EOB-MRI: Gadolinium-ethoxybenzyl-diethylenetriamine pentaacetic acid-enhanced magnetic resonance imaging
CT: Computed tomography
US: Ultrasonography
FDG-PET: [^18^F]-fluorodeoxyglucose positron emission tomography
